# Genetic Characterization of the First Case of Lumpy Skin Disease in Singapore, 2022

**DOI:** 10.3390/vetsci12111108

**Published:** 2025-11-20

**Authors:** Eileen Y. Koh, Adrian K. S. Tan, Yin Cheong Aden Ip, Clara Lau, Jasmine Ong, Oi Wing Ng, Jing Chen, Christine Lee, Suria Fabbri, Juline Chua, Samyuktha Balakumar, Kelvin Ho, Wai Kwan Wong, Brian Z. Y. Tan, Charlene Judith Fernandez, Siow Foong Chang, Him Hoo Yap

**Affiliations:** Animal and Veterinary Service, National Parks Board, Singapore Botanic Gardens, 1 Cluny Road, Singapore 259569, Singapore; adrian_tan@nparks.gov.sg (A.K.S.T.); jasmine_ong@nparks.gov.sg (J.O.); chen_jing@nparks.gov.sg (J.C.); wong_wai_kwan@nparks.gov.sg (W.K.W.);

**Keywords:** Lumpy Skin Disease, Singapore, outbreak detection, next generation sequencing, molecular detection

## Abstract

Lumpy Skin Disease (LSD) is a transboundary viral disease of wild and domesticated ruminants, and notifiable to the World Organisation for Animal Health (WOAH). LSD has spread rapidly in the cattle population across the African, European and Asian continents since 1929. Following recent outbreaks in the Southeast Asia region, Singapore observed its first occurrence of LSD in a local dairy cattle farm in March 2022. LSD was confirmed in dairy cattle exhibiting clinical signs with quantitative real-time PCR and sequencing with MinION Nanopore. Analyses of the assembled whole viral genomes also revealed high phylogenetic relatedness to LSD recombinant strains. It remains unclear how the virus was introduced into Singapore, given the absence of known vectors and strict import regulations in place. This highlights the importance of biosurveillance, and laboratory diagnostic readiness to manage outbreaks and prevent the spread of transboundary diseases.

## Simple Summary

This study documented and characterised Singapore’s first outbreak of Lumpy Skin Disease (LSD), a transboundary viral disease affecting cattle that poses significant threats to livestock production and trade. In March 2022, the National Parks Board investigated an outbreak at a local dairy farm where cattle exhibited characteristic dermatological lesions. The laboratory investigation employed real-time PCR and whole genome sequencing to analyse samples from affected animals. Results showed that 11 of 13 samples from one farm tested positive for the LSD virus, whilst a neighbouring farm remained unaffected. Phylogenetic analysis revealed that the Singapore LSD strain clustered with recombinant variants circulating in Asia, particularly those from Thailand, China, and Russia, suggesting regional transmission patterns. The outbreak was successfully contained through the immediate implementation of movement restrictions, vector control measures, and enhanced biosecurity protocols, with clinical signs resolving within one month without requiring vaccination or culling. This disease investigation demonstrates the critical importance of robust disease surveillance systems and rapid diagnostic capabilities in managing transboundary animal diseases. The findings contribute valuable epidemiological data for understanding LSD transmission dynamics in Southeast Asia and inform evidence-based strategies for preventing future incursions, thereby protecting livestock industries, and maintaining food security.

## 1. Introduction

Lumpy Skin Disease (LSD) is a vector-borne viral disease which affects the production and trade of cattle, buffaloes, and some wild ruminants [1]. LSD can also be transmitted through direct contact between infected and susceptible animals [2]. This disease is caused by the genus *Capripoxvirus* of the family *Poxviridae*, closely related Goatpox and Sheeppox. Notably, LSD is a WOAH-notifiable disease due to its rapid spread and consequential substantial economic losses [3,4]. The genome of the LSD virus (LSDv) comprises a double-stranded linear DNA of around 150 kilobases, encased within a lipid envelope [5].

LSD emerged from the African continent and subsequently spread into countries in Europe and Asia [6]. LSDv is primarily transmitted through haematophagous vectors, such as hard ticks (*Rhipicephalus* spp. and *Amblyomma* spp.) [7], mosquitoes (*Aedes* spp.) [8] and stable flies (*Stomoxys* spp.) [9], which could facilitate rapid transboundary spread under favourable environmental conditions. Furthermore, several factors including bioclimatic conditions, land type, and population density have been predicted to influence LSD outbreaks [10]. Depending on the virus strain, immune status of the hosts, and abundance of mechanical arthropod vectors, LSD exhibits a comparatively lower mortality rate (≤10%) and varied morbidity rates (up to 85%) than other transboundary diseases [11].

The rapid disease occurrences into previously disease-free countries [12,13,14,15,16] highlights the importance of recognising the pathways and limitations of the transmission of LSD. Global climate changes, the illegal trade of animals and animal products, fluctuations of livestock movement, and periods of civil unrest had been previously described to contribute to increased outbreaks of LSD [6]. Conversely, the limited knowledge of LSD transmission could apply to geographically isolated countries [17] and lack of documented outbreaks in wildlife for a comprehensive scope in wildlife epidemiology [18]. Thus, the impact of these developments upon livestock management and concerns of food security have elicited significant attention among the affected and previously disease-free countries [6].

In the event of an LSD outbreak, culling of all susceptible and clinically infected animals is recommended [19]. While live attenuated LSD vaccines are commonly employed by countries to confer protection to the ruminants in the event of an outbreak, the challenges to protect and manage cattle are compounded by the issue of vaccine quality, efficacy, price and side effects [20]. Also, the absence of safe and efficient vaccines thus far can be attributed to LSDv’s innate immune escape mechanism. Moreover, the emergence of recombinant vaccine-like LSDv field strains has complicated the detection of true field strain infection [21,22], given the sequence similarities between the target region(s) of the homologous vaccine and recombinant strains. This intricate interplay between the LSD and the vaccine limitations underscores the urgency of comprehensive research and international collaboration to address the LSDv challenges effectively.

In February 2022, the National Parks Board (NParks), Singapore, was alerted to cattle from a local dairy cattle farm displaying clinical dermatological signs, i.e., multiple skin nodules disseminated across various parts of the body, suggestive of LSD. Additionally, other clinical signs such as intermittent coughing and ulceration of the nasum were also observed by the attending veterinarian. The present work describes the disease investigation, detection and molecular characterisation of the first incursion of LSDv in a local dairy cattle farm in Singapore in 2022.

## 2. Methods

### 2.1. Outbreak Investigation and Sample Collection

On 28 February 2022, the authorities were alerted to a possible LSD outbreak in local dairy cattle (*Bos taurus*) via industry feedback. The two dairy farms were located closely to each other, with 58 (Farm 1) and 55 (Farm 2) cattle, respectively. Both farms had self-replacing herds with no reported import of cattle. Disease investigation was then carried out on the affected farms, with the respective owners interviewed. The cattle in Farm 1 were in a satisfactory body condition (2.5 to 3 out of 5) and observed to be eating and drinking; cattle in Farm 2 were observed to be in a good body condition (3 to 4 out of 5) and eating well.

A total of 22 nasal swabs and 2 skin swabs from ulcerated skin nodules were collected from cattle presented with dermatological and clinical signs suggestive of LSD, out of the 113 (14.2%) cattle from the two farms by the field-investigating officers, who were official veterinarians from the National Parks Board. The dry swabs of the nasal cavity and open lesions were kept in virus transport media (VTM; Puritan^®^ UniTranz-RT^®^ Transport System, Guilford, MN, USA) under cool conditions, and subsequently sent to the Centre of Animal and Veterinary Sciences, Singapore for laboratory diagnostic analyses on the same day.

### 2.2. Sample Extraction and Molecular Detection

Nucleic acids were extracted with the IndiMag^®^ Pathogen Kit (Indical Bioscience GmbH, Leipzig, Germany), according to the manufacturer’s guidelines. Real-time PCR (RT-PCR) targeting the intracellular mature virion envelope protein P32 was performed on the samples [23], where Ct values of <45 cycles were considered indicative of potential LSDv detection.

### 2.3. Whole Genome Sequencing Using Oxford Nanopore Technology and Illumina Paired End Sequencing

LSD-positive samples were sequenced on the Oxford Nanopore Technology (ONT) MinION Mk1b sequencer (Oxford Nanopore Technologies Inc., Oxford, UK) and Illumina (ILM) iSEQ 100 system (Illumina Inc., San Diego, CA, USA) by paired-end sequencing. In brief, the process involved the preparation of a MinION nanopore library preparation using the Native Barcoding (EXP-NBD104) (Oxford Nanopore Technologies Inc., Oxford, United Kingdom) and Ligation Sequencing Kits (SQK-LSK109) (Oxford Nanopore Technologies Inc., Oxford, UK) for total DNA. The library was subsequently loaded onto a Flo-MIN106 R9.4.1 flow cell and sequenced for 24 h. All runs were initiated with MinKNOW (v21.11.7) and base-calling was performed using Guppy (v6.5.7), using the high accuracy model. For the Illumina library, the Illumina DNA Prep kit was used with NEXTERA DNA CD Indexes (Illumina Inc., San Diego, CA, USA). The library was subsequently sequenced on the iSEQ 100 system (Illumina Inc., San Diego, CA, USA) and basecalling was performed with the fastq generator module on board.

A total of 1552 and 424 reads were obtained, respectively, from the ONT and ILM runs. The reads were mapped to the reference LSD genome (NC_003027.1) with Minimap (v2.24) and bwa (v0.17.7). Resultant alignment BAM files from both mappings were combined and a consensus sequence was generated using samtools consensus (v1.17). The resulting sequence was assigned the designation “SG/NParks/SG/A-MAM-2022-03-00038” (NCBI accession number PX378906).

### 2.4. Sequence and Phylogenetic Analysis

A total of 82 complete genome sequences of field and vaccine LSD, Sheeppox (NC_004002.1) and Goatpox (NC_004003.1) viruses were retrieved from GenBank (https://www.ncbi.nlm.nih.gov/, accessed on 10 September 2025). The maximum likelihood method was used to build 800 bootstrap trees, and a consensus tree was aggregated to summarise the relationship of the “SG/NParks/A-MAM-2022-03-00038” virus sequence with respect to the broader spectrum of *Capripoxviruses* species using RAxML-NG (V1.1.0) and visualised with MEGA X version 10.2.6 [24]. Sheeppox (NC_004002.1) and Goatpox (NC_004003.1) were used as the root for the phylogenetic tree.

### 2.5. Recombination Analysis

An alignment consisting of four LSDv complete genomes from Singapore, Thailand (ON152411), China (MW355944) and Vietnam (MZ577075) were constructed and analysed on the RDP4 software with the default settings. The Singapore LSDv strain will be considered a potential recombinant if at least three out of seven detection methods presented a *p*-value of less than 0.05 [25].

## 3. Results

### 3.1. Clinical Manifestations of LSDv

**Figure 1:**
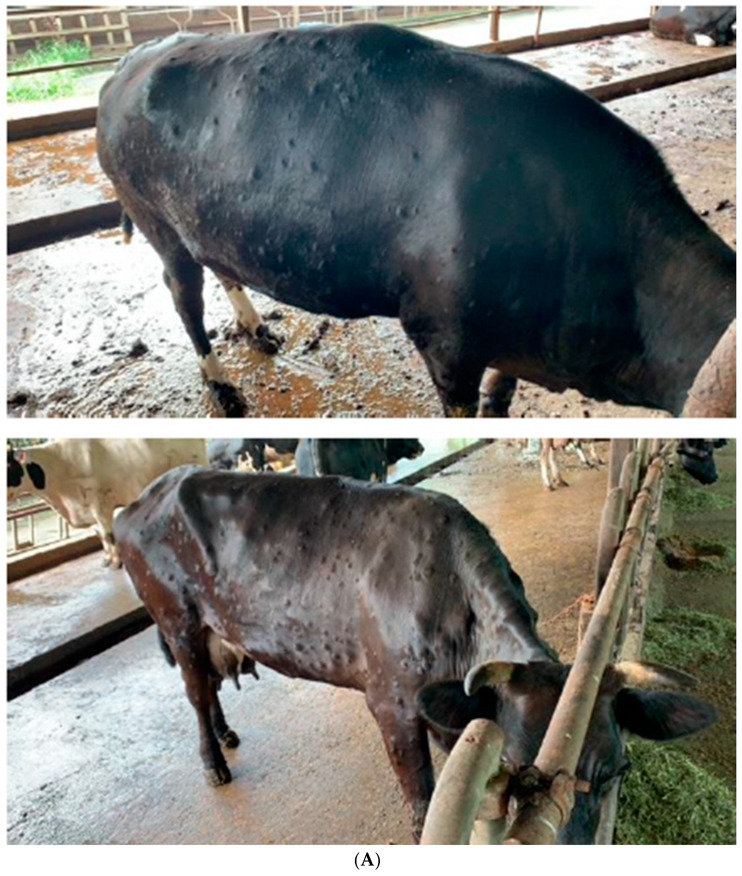

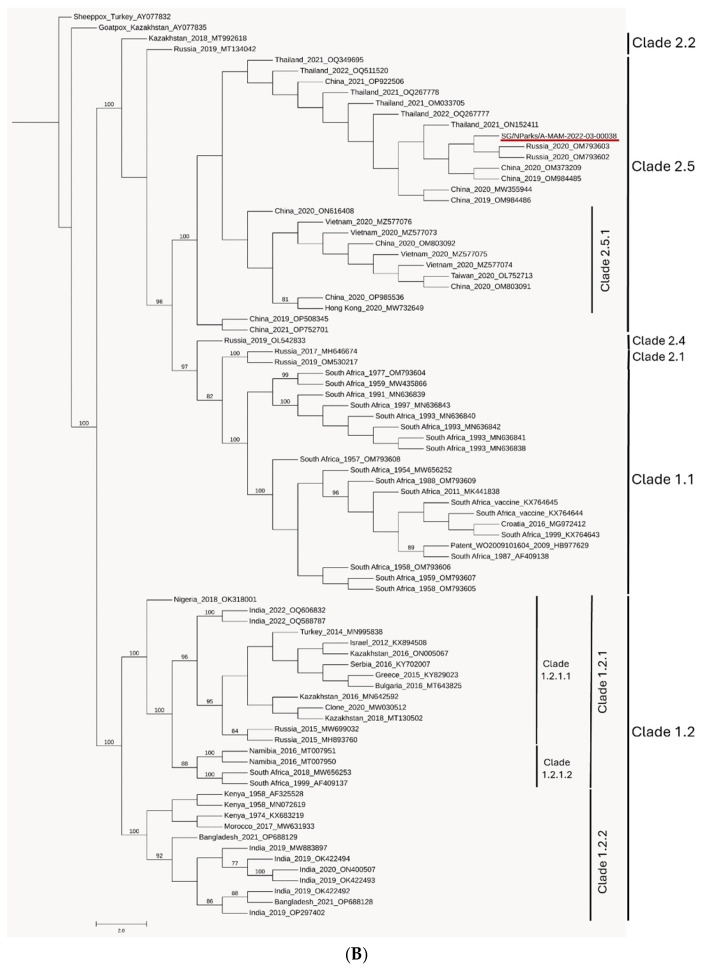
(**A**) Cattle showing clinical signs of Lumpy Skin Disease with nodular skin lesions covering the entire body. Raised nodular lesions of 2–5 cm in diameter were observed on the affected cattle. (**B**) Cladogram of full-length (approximately 150,000 bp) capripoxvirus genomes. Distinct clades of Lumpy Skin Disease virus could be seen with the sequences, referenced from [26]. Within Clades 1.1 (historical South African) and 1.2 (Pan-African/Eurasian), vaccine-based and classical wild-type LSDv form separate clades. For Clade 2.5 (recombinant strains), sequences could be distinguished by their geographical origins. The sequence identified in this study is labelled as “Singapore/NParks/LSDV/A-MAM-2022-02-00038” and underlined in red (NCBI accession number PX378906). The phylogenetic tree was rooted to Sheeppox (AY077832).

Skin lesions or nodules, varying in severity from mild (fewer than five) to severe (more than 10) were observed on different body parts of the affected cattle (Figure 1A). Notably, these manifestations were exclusively documented from one of the two farms, with no death cases reported. One bull displayed multiple ulcerated nodular skin lesions and exhibited signs of depression, while a cow was presented with ulceration in the nose from Farm 1. Two cattle in Farm 2 were presented with intermittent coughing. The herds were not vaccinated before this outbreak event. The owners of the affected farm were issued with an isolation order to restrict animal movements both within and beyond their premises to restrict animal movement to safeguard against potential spread of the disease. As part of the comprehensive disease investigation efforts, the farm received veterinary and farm management support from NParks through proactive measures encompassing vector and environmental controls. Vector control strategies, such as that for the farms to spray insecticide on a daily basis and the drainage of stagnant waters, together with routine husbandry protocols, were considered as licencing conditions for biosecurity. As the clinical signs eventually resolved, the herds were not vaccinated post-infection.

### 3.2. Molecular Detection of LSDv

Among the submitted samples from both farms, eleven (i.e., two skin and nine nasal swabs) out of thirteen samples from Farm 1 yielded detection for LSDv by RT-PCR (Table 1; Ct values ranged from 24.10 to 30.49). Conversely, LSDv was not detected from all eleven samples from Farm 2 (i.e., 11 nasal swabs) and were hence excluded from further analysis. Three weeks after the initial episode, skin swabs and blood samples from the coccygeal vein were collected from Farm 1 and were again tested by RT-PCR (Table 1; Ct 26.98 to 38.5) as follow-up to the disease management. Hereafter, no further LSDv events were observed, the isolation order was lifted on 19 July 2022, and the farm was placed under passive surveillance for LSDv.

### 3.3. Whole Genome Sequencing and Phylogenetic Analysis of LSDv Genome

Alignment of the assembled 150,773 bp genome with publicly available LSDv sequences showed that SG/NParks/A-MAM-2022-03-00038 clustered to sequences in Clade 2.5 reported between 2020 and 2021 from Russia, China Xinjiang, and Thailand, but not to Clade 2.5.1 reported predominantly in East Asia (i.e., China, Taiwan) (Figure 1B). Most of the predicted events from RDP4 also did not support that SG/NParks/A-MAM-2022-03-00038 had undergone genetic changes, where no significant *p*-values (i.e., *p* > 0.05) were recorded from five out of the seven methods, and is similar to previously reported strains in Clade 2.5.

On 9 March 2022, Singapore submitted an official notification of the LSD occurrence to the WOAH [18].

## 4. Discussion

LSD, once only endemic in the African continent, was introduced to the Middle East during the last century. It subsequently spread across Europe and Asia, posing a significant challenge to livestock management and threatened food security [6]. Following the emergence of LSDv recombination in Russia [27,28], the disease had since spread across East and Southeast Asia, where over 90,000 cases and a higher-than-average mortality rate of 2.7% have been reported in the latter region [29]. The occurrence of capripoxvirus outbreaks has shown an upward trend since 2000 [30], aligning with the improvements in the detection and sequencing technologies, leading to the availability of more full-length viral genomes [21,31,32]. Recent advancements in sequencing technologies have also enabled the monitoring of whole LSDv genomes, aiding in the identification of genetic changes and the flagging of potentially novel recombinant viruses that may explain its spread, virulence and host establishment [16,33,34]. With the confirmation of the presence of LSDv by RT-PCR and WGS, the Singapore LSDv strain showed similarities to recombinant strains circulating within Asia [12,16,33,35].

Poxviruses evolve via slow genetic drift and rapid genetic shifts through recombination [27]. The existence of multiple LSDv strains within proposed LSDv clades [26] had actually adapted over time to the local animal populations, and potentially developed unique genetic signatures specific to their respective regions [36]. These insights shed light on factors influencing both the virulence and evolution of the capripoxviruses in different host species [37], and could explain the differences, in terms of morbidity and mortality, observed. Studies have also suggested that independent clustering of the LSDv strain could either present unique evolutionary characteristics or exhibit similar genetic patterns to the recombinant vaccine-like strain [12,21].

LSD vaccines are produced in attenuated forms, which allow for the generation of relatively random mutations throughout the viral genome due to altered selection pressures [38]. Despite the availability and practice of vaccination, horizontal transmission of LSDv through a vaccine-derived virulent recombinant strain was not characterised until recently [22,35]. This could render traditional diagnostic tools like the differentiation between infected and vaccinated animal (DIVA) PCR assays to be less effective [39]. Hence, refinement of the DIVA assays is essential to improve specificity and sensitivity for distinguishing between the field and vaccine-like recombinant LSDv strains.

The source of infection is unknown from this disease investigation and the clinical signs on affected cattle were resolved about a month later. Given LSD’s incubation period of 6 to 26 days [16,40], infections in the non-vaccinated local herd could have occurred for a period of time before the manifestation of clinical signs or vaccination implementation. Contributing factors to the LSDv spread include warm humid agro-climates, the introduction of new animals into the herd, and communal feeding sources [41]. While non-vector [22] transmission of LSDv are plausible, the potential introduction of LSDv through imported contaminated animal feed or articles harbouring inactivated LSD viral particles necessitates further elucidation. Control strategies for LSDv must continually undergo review for the development of holistic considerations toward risk analyses, in addition to the traditional vector control and monitoring programmes.

## 5. Conclusions

It is prudent for global authorities to vigilantly monitor the potential spread and spill-over effects of LSDv to other non-ruminant animals, such as wild banteng [42], giraffes [43] and camels [44] as these animals could serve as potential reservoirs. As such, local stakeholders were informed to continuously monitor ruminant and non-ruminant animals for clinical signs of LSD as part of enhanced passive surveillance. These instances highlight the importance of robust disease biosurveillance systems and necessitate the implementation of strict border control programmes and a comprehensive framework for effective control measures.

This study demonstrates the pivotal role played by disease biosurveillance, emphasising the imperative to establish a well-coordinated strategy between field epidemiology and laboratory diagnostics. It is imperative to not only consider immediate containment but also to delve into broader strategies encompassing vaccination regimes, vector control protocols, and improved diagnostic frameworks. The complexities of such disease outbreak scenarios require a holistic management approach to minimise potential risks and ensure the well-being of both livestock and our natural ecosystems.

Since this incident, no further cases have been reported in Singapore since the last report to WOAH on 21 July 2022 [18]. This step is crucial for committing to transparency and fosters strong collaboration in addressing future disease outbreaks, thus, safeguarding both animal health and welfare. 

## Figures and Tables

**Table 1:** Summary of real-time PCR of LSD detection. Only samples collected from Farm 1 were positive for LSD detected by real-time PCR. Ct values of positive sample (<45 cycles) from the real-time PCR were reported to two decimal places; negative samples were reported as ‘Undetermined’. Positive control used was the LSDv Neethling strain nucleic acid (source: The Pirbright Institute Catalogue Number NVRL-CPV-LS-NA-001); negative control was PCR reagent mixture only. Nsb = nasal swab.

Farm 1	Samples	LSD Real-Time PCR [20]	
		Detection	Ct Values
A-MAM-2022-03-00038 *Collection date:* *9 March 2022*	Nsb 1	Detected	35.58
	Nsb 2	Detected	25.51
	Nsb 3	Detected	30.50
	Nsb 4	Detected	32.32
	Nsb 5	Detected	36.81
	Nsb 6	Detected	38.69
	Nsb 7	Detected	26.52
	Nsb 8	Detected	36.26
	Nsb 9	Not detected	Undetermined
	Nsb 10	Detected	33.05
	Nsb 11	Not detected	Undetermined
	Skin swab 1	Detected	26.05
	Skin swab 2	Detected	24.12
*Collection date:* *23 March 2022*	Skin swab 1	Detected	32.70
	Skin swab 2	Detected	26.98
	Skin swab 3	Detected	27.46
	Skin swab 4	Detected	40.88
	Skin swab 5	Detected	33.35
	Skin swab 6	Detected	39.37
	EDTA Blood	Detected	38.5

## Data Availability

The original data presented in the study are openly available in NCBI GenBank at accession number PX378906.

## References

[1] World Organisation for Animal Health (2023). Lumpy Skin Disease. Manual of Diagnostic Tests and Vaccines for Terrestrial Animals.

[2] Sprygin A., Pestova Y., Wallace D.B., Tuppurainen E., Kononov A.V. (2019). Transmission of lumpy skin disease virus: A short review. Virus Res..

[3] Molla W., de Jong M.C.M., Gari G., Frankena K. (2017). Economic impact of lumpy skin disease and cost effectiveness of vaccination for the control of outbreaks in Ethiopia. Prev. Vet. Med..

[4] Limon G., Gamawa A.A., Ahmed A.I., Lyons N.A., Beard P.M. (2020). Epidemiological Characteristics and Economic Impact of Lumpy Skin Disease, Sheeppox and Goatpox Among Subsistence Farmers in Northeast Nigeria. Front. Vet. Sci..

[5] Tulman E.R., Afonso C.L., Lu Z., Zsak L., Kutish G.F., Rock D.L. (2001). Genome of lumpy skin disease virus. J. Virol..

[6] Azeem S., Sharma B., Shabir S., Akbar H., Venter E. (2022). Lumpy skin disease is expanding its geographic range: A challenge for Asian livestock management and food security. Vet. J..

[7] Tuppurainen E.S., Stoltsz W.H., Troskie M., Wallace D.B., Oura C.A., Mellor P.S., Coetzer J.A., Venter E.H. (2011). A potential role for ixodid (hard) tick vectors in the transmission of lumpy skin disease virus in cattle. Transbound. Emerg. Dis..

[8] Paslaru A.I., Maurer L.M., Vögtlin A., Hoffmann B., Torgerson P.R., Mathis A., Veronesi E. (2022). Putative roles of mosquitoes (*Culicidae*) and biting midges (*Culicoides* spp.) as mechanical or biological vectors of lumpy skin disease virus. Med. Vet. Entomol..

[9] Issimov A., Kutumbetov L., Orynbayev M.B., Khairullin B., Myrzakhmetova B., Sultankulova K., White P.J. (2020). Mechanical Transmission of Lumpy Skin Disease Virus by *Stomoxys* spp. (*Stomoxys calsitrans*, *Stomoxys sitiens*, *Stomoxys indica*), Diptera: Muscidae. Animals.

[10] Li Y., An Q., Sun Z., Gao X., Wang H. (2023). Risk Factors and Spatiotemporal Distribution of Lumpy Skin Disease Occurrence in the Asian Continent during 2012–2022: An Ecological Niche Model. Transbound. Emerg. Dis..

[11] Akther M., Akter S.H., Sarker S., Aleri J.W., Annandale H., Abraham S., Uddin J.M. (2023). Global Burden of Lumpy Skin Disease, Outbreaks, and Future Challenges. Viruses.

[12] Lu G., Xie J., Luo J., Shao R., Jia K., Li S. (2021). Lumpy skin disease outbreaks in China, since 3 August 2019. Transbound. Emerg. Dis..

[13] Badhy S.C., Chowdhury M.G.A., Settypalli T.B.K., Cattoli G., Lamien C.E., Fakir M.A.U., Akter S., Osmani M.G., Talukdar F., Begum N. (2021). Molecular characterization of lumpy skin disease virus (LSDV) emerged in Bangladesh reveals unique genetic features compared to contemporary field strains. BMC Vet. Res..

[14] Tran H.T.T., Truong A.D., Dang A.K., Ly D.V., Nguyen C.T., Chu N.T., Hoang T.V., Nguyen H.T., Nguyen V.T., Dang H.V. (2021). Lumpy skin disease outbreaks in vietnam, 2020. Transbound. Emerg. Dis..

[15] Arjkumpa O., Suwannaboon M., Boonrawd M., Punyawan I., Laobannu P., Yantaphan S., Bungwai A., Ponyium V., Suwankitwat N., Boonpornprasert P. (2021). First emergence of lumpy skin disease in cattle in Thailand, 2021. Transbound. Emerg. Dis..

[16] Flannery J., Shih B., Haga I.R., Ashby M., Corla A., King S., Freimanis G., Polo N., Tse A.C., Brackman C.J. (2022). A novel strain of lumpy skin disease virus causes clinical disease in cattle in Hong Kong. Transbound. Emerg. Dis..

[17] Hall R.N., Torpy J.R., Nye R., Zalcman E., Cowled B.D. (2023). A quantitative risk assessment for the incursion of lumpy skin disease virus into Australia via long-distance windborne dispersal of arthropod vectors. Prev. Vet. Med..

[18] World Organisation for Animal Health (2024). Lumpy Skin Disease, Singapore—Notification Report.

[19] Tuppurainen E., Alexandrov T., Beltran-Alcrudo D. (2017). Lumpy Skin Disease Field Manual—A Manual for Veterinarians.

[20] Tuppurainen E., Dietze K., Wolff J., Bergmann H., Beltran-Alcrudo D., Fahrion A., Lamien C.E., Busch F., Sauter-Louis C., Conraths F.J. (2021). Review: Vaccines and Vaccination against Lumpy Skin Disease. Vaccines.

[21] Sprygin A., Babin Y., Pestova Y., Kononova S., Wallace D.B., Van Schalkwyk A., Byadovskaya O., Diev V., Lozovoy D., Kononov A. (2018). Analysis and insights into recombination signals in lumpy skin disease virus recovered in the field. PLoS ONE.

[22] Aleksandr K., Olga B., David W.B., Pavel P., Yana P., Svetlana K., Alexander N., Vladimir R., Dmitriy L., Alexander S. (2020). Non-vector-borne transmission of lumpy skin disease virus. Sci. Rep..

[23] Bowden T.R., Babiuk S.L., Parkyn G.R., Copps J.S., Boyle D.B. (2008). Capripoxvirus tissue tropism and shedding: A quantitative study in experimentally infected sheep and goats. Virology.

[24] Kumar S., Stecher G., Li M., Knyaz C., Tamura K. (2018). MEGA X: Molecular Evolutionary Genetics Analysis across Computing Platforms. Mol. Biol. Evol..

[25] Martin D.P., Murrell B., Golden M., Khoosal A., Muhire B. (2015). RDP4: Detection and analysis of recombination patterns in virus genomes. Virus Evol..

[26] Breman F.C., Haegeman A., Krešić N., Philips W., De Regge N. (2023). Lumpy Skin Disease Virus Genome Sequence Analysis: Putative Spatio-Temporal Epidemiology, Single Gene versus Whole Genome Phylogeny and Genomic Evolution. Viruses.

[27] Sprygin A., Artyuchova E., Babin Y., Prutnikov P., Kostrova E., Byadovskaya O., Kononov A. (2018). Epidemiological characterization of lumpy skin disease outbreaks in Russia in 2016. Transbound. Emerg. Dis..

[28] Kononov A., Byadovskaya O., Kononova S., Yashin R., Zinyakov N., Mischenko V., Perevozchikova N., Sprygin A. (2019). Detection of vaccine-like strains of lumpy skin disease virus in outbreaks in Russia in 2017. Arch. Virol..

[29] Wilhelm L., Ward M.P. (2023). The Spread of Lumpy Skin Disease Virus across Southeast Asia: Insights from Surveillance. Transbound. Emerg. Dis..

[30] Tuppurainen E.S.M., Venter E.H., Shisler J.L., Gari G., Mekonnen G.A., Juleff N., Lyons N.A., De Clercq K., Upton C., Bowden T.R. (2017). Review: Capripoxvirus Diseases: Current Status and Opportunities for Control. Transbound. Emerg. Dis..

[31] Eltom K.H., Althoff A.C., Hansen S., Böhlken-Fascher S., Yousif A., El-Sheikh H.A., ElWakeel A.A., Elgamal M.A., Mossa H.M., Aboul-Soud E.A. (2021). Differentiation of Capripox Viruses by Nanopore Sequencing. Vaccines.

[32] Wei Y.-R., Ma W.-G., Wang P., Wang W., Su X.-H., Yang X.-Y., Mi X.-Y., Wu J.-Y., Huang J. (2023). Retrospective genomic analysis of the first Lumpy skin disease virus outbreak in China (2019). Front. Vet. Sci..

[33] Koirala P., Meki I.K., Maharjan M., Settypalli B.K., Manandhar S., Yadav S.K., Cattoli G., Lamien C.E. (2022). Molecular Characterization of the 2020 Outbreak of Lumpy Skin Disease in Nepal. Microorganisms.

[34] Krotova A., Byadovskaya O., Shumilova I., van Schalkwyk A., Sprygin A. (2022). An in-depth bioinformatic analysis of the novel recombinant lumpy skin disease virus strains: From unique patterns to established lineage. BMC Genom..

[35] Sprygin A., Pestova Y., Bjadovskaya O., Prutnikov P., Zinyakov N., Kononova S., Ruchnova O., Lozovoy D., Chvala I., Kononov A. (2020). Evidence of recombination of vaccine strains of lumpy skin disease virus with field strains, causing disease. PLoS ONE.

[36] Xie S., Cui L., Liao Z., Zhu J., Ren S., Niu K., Li H., Jiang F., Wu J., Wang J. (2024). Genomic analysis of lumpy skin disease virus asian variants and evaluation of its cellular tropism. NPJ Vaccines.

[37] Biswas S., Noyce R.S., Babiuk L.A., Lung O., Bulach D.M., Bowden T.R., Boyle D.B., Babiuk S., Evans D.H. (2020). Extended sequencing of vaccine and wild-type capripoxvirus isolates provides insights into genes modulating virulence and host range. Transbound. Emerg. Dis..

[38] Fenner F., Cairns J., Burnet F.M., Stanley W.M. (1959). Variation in Virulence in Relation to Adaptation to New Hosts. The Viruses, Animal Viruses.

[39] Byadovskaya O., Pestova Y., Kononov A., Shumilova I., Kononova S., Nesterov A., Babiuk S., Sprygin A. (2021). Performance of the currently available DIVA real-time PCR assays in classical and recombinant lumpy skin disease viruses. Transbound. Emerg. Dis..

[40] Sohier C., Haegeman A., Mostin L., De Leeuw I., Campe W.V., De Vleeschauwer A., Tuppurainen E.S.M., van den Berg T., De Regge N., De Clercq K. (2019). Experimental evidence of mechanical lumpy skin disease virus transmission by *Stomoxys calcitrans* biting flies and *Haematopota* spp. horseflies. Sci. Rep..

[41] Gari G., Waret-Szkuta A., Grosbois V., Jacquiet P., Roger F. (2010). Risk factors associated with observed clinical lumpy skin disease in Ethiopia. Epidemiol. Infect..

[42] Porco A., Chea S., Sours S., Nou V., Groenenberg M., Agger C., Tum S., Chhuon V., Sorn S., Hong C. (2023). Case report: Lumpy skin disease in an endangered wild banteng (*Bos javanicus*) and initiation of a vaccination campaign in domestic livestock in Cambodia. Front. Vet. Sci..

[43] Dao T.D., Tran L.H., Nguyen H.D., Hoang T.T., Nguyen G.H., Tran K.V.D., Nguyen H.X., Van Dong H., Bui A.N., Bui V.N. (2022). Characterization of Lumpy skin disease virus isolated from a giraffe in Vietnam. Transbound. Emerg. Dis..

[44] Kumar R., Godara B., Chander Y., Kachhawa J.P., Dedar R.K., Verma A., Riyesh T., Pal Y., Barua S., Tripathi B.N. (2023). Evidence of lumpy skin disease virus infection in camels. Acta Trop..

